# The Complexity of H-wave Amplitude Fluctuations and Their Bilateral Cross-Covariance Are Modified According to the Previous Fitness History of Young Subjects under Track Training

**DOI:** 10.3389/fnhum.2017.00530

**Published:** 2017-11-01

**Authors:** Maria E. Ceballos-Villegas, Juan J. Saldaña Mena, Ana L. Gutierrez Lozano, Francisco J. Sepúlveda-Cañamar, Nayeli Huidobro, Elias Manjarrez, Joel Lomeli

**Affiliations:** ^1^Sección de Posgrado e Investigación, Laboratorio de Neurofisiología Humana y Control Motor, Escuela Superior de Medicina, Instituto Politécnico Nacional, Mexico City, Mexico; ^2^Escuela de Quiropráctica, Universidad Estatal del Valle de Ecatepec, Ecatepec de Morelos, Mexico; ^3^UMAA7, IMSS, San Pedro Garza, Mexico; ^4^Instituto de Fisiología, Benemérita Universidad Autónoma de Puebla, Puebla, Mexico

**Keywords:** H-wave, amplitude fluctuation, complexity, alpha-motoneuron, cross-covariance, fractal dimension

## Abstract

The Hoffmann reflex (H-wave) is produced by alpha-motoneuron activation in the spinal cord. A feature of this electromyography response is that it exhibits fluctuations in amplitude even during repetitive stimulation with the same intensity of current. We herein explore the hypothesis that physical training induces plastic changes in the motor system. Such changes are evaluated with the fractal dimension (FD) analysis of the H-wave amplitude-fluctuations (H-wave FD) and the cross-covariance (CCV) between the bilateral H-wave amplitudes. The aim of this study was to compare the H-wave FD as well as the CCV before and after track training in sedentary individuals and athletes. The training modality in all subjects consisted of running three times per week (for 13 weeks) in a concrete road of 5 km. Given the different physical condition of sedentary vs. athletes, the running time between sedentary and athletes was different. After training, the FD was significantly increased in sedentary individuals but significantly reduced in athletes, although there were no changes in spinal excitability in either group of subjects. Moreover, the CCV between bilateral H-waves exhibited a significant increase in athletes but not in sedentary individuals. These differential changes in the FD and CCV indicate that the plastic changes in the complexity of the H-wave amplitude fluctuations as well as the synaptic inputs to the Ia-motoneuron systems of both legs were correlated to the previous fitness history of the subjects. Furthermore, these findings demonstrate that the FD and CCV can be employed as indexes to study plastic changes in the human motor system.

## Introduction

It is very well known that the Hoffmann reflex (H-wave) is susceptible to plastic changes in animals (Wolpaw, 1997; Wolpaw and Tennissen, 2001) and humans (Mazzocchio et al., 2006; Meunier et al., 2007; Lamy et al., 2012). Modulation of the vertebrate H-wave could involve plasticity at multiple sites, including the synaptic contacts in both interneurons and alpha-motoneurons (Thompson and Wolpaw, 2014). Additionally, the intrinsic properties of firing threshold and conduction velocity of alpha-motoneurons may affect the H-wave amplitude (Gardiner et al., 2006).

Plasticity may also involve changes in the fractal complexity of the synaptic response of the alpha-motoneurons (Werner, 2010). In fact, Nozaki and collaborators found that the amplitude fluctuation of H-wave in healthy subjects was fractal with a strong time-correlation (Nozaki et al., 1995). In contrast, the M-wave sequence had a significantly weaker time-correlation. These authors suggested that the fractal correlation found in the human H-wave was produced at the synaptic connections from Ia-afferents to alpha-motoneurons in the spinal cord.

Higuchi's fractal dimension is useful for the complexity analysis of a variety of biological signals. For example, this method has been used in the analysis of EEG (Ferenets et al., 2006; Sabeti et al., 2009; Ahmadlou et al., 2010, 2012; Khoa et al., 2012b; Bachmann et al., 2013, 2014; Wang and Sourina, 2013; Zhang et al., 2014), MEG (Gomez et al., 2009), ECG (Don et al., 2013), and EMG signals (Khoa et al., 2012a; Cukic et al., 2013). However, to our knowledge, there are no reports on Higuchi's fractal dimension (FD) for H-wave recordings. Applying this analytical tool to the present data revealed possible changes in the connectivity patterns of the reflex pathways in the spinal cord.

The first aim of the present study was to explore plastic changes in the complexity of the recruitment order of motor units in both medial gastrocnemius muscles of a group of young athletes. Such changes were evaluated by determining the H-wave amplitude fluctuations after a light training program. The data can be used to understand the mechanism involved in physiological adaptation to exercise. Higuchi's method was employed to calculate the FD for the amplitude fluctuations of 130 successive H-waves recorded from the right and left medial gastrocnemius muscles before and after track training. As a control, we explored whether similar changes in the complexity of the H-wave amplitude fluctuations occurred in sedentary individuals. The second aim of the study was to examine whether the CCV between bilateral H-waves exhibited comparable changes in the subjects before and after training. In humans (Mezzarane and Kohn, 2002) and cats (Manjarrez et al., 2005), it has been demonstrated that the simultaneous stimulation of Ia afferents in both legs produces bilateral H-waves with a significant CCV in their amplitude fluctuations. By employing the same software used by these research groups, we herein calculated the CCV between bilateral H-wave amplitudes.

The training modality in all subjects consisted of running three times per week (for 13 weeks) in a concrete road of 5 km. The justification to employ this training modality was that it was not too demanding for the sedentary subjects and not too simple for the athletes. Similar training modalities are commonly employed for beginning runners to prevent injuries and to obtain health benefits (e.g., see handbook by Macneill and The Sport Medicine Council of British Columbia, 2012). The functional implications of the results after this training modality are that exercise for beginners would be enough to produce plastic changes associated with the H-wave neuronal pathways.

Our study will reinforce the hypothesis that physical training induces plastic changes in the motor system, in particular in the neuronal pathways related to the amplitude fluctuations of the H-wave.

## Methods

### Experimental subjects

Bilateral H-waves were recorded on 16 young subjects without any clinically detectable neurological damage. Eight subjects had no prior sports background (sedentary individuals); i.e., they did not practice any sports activity before their participation in the experiments. The other eight subjects had practiced track and field and other sports for several years (athletes). The type of track and field and sports practiced by these athletes is illustrated in Table 1. Sedentary individuals: 19.37 ± 1.3 years; 61.52 ± 14.36 kg; 1.63 ± 0.07 m. Athletes: 20.25 ± 1.83 years; 63.44 ± 13.83 kg; 1.67 ± 0.87 m.

**Table 1:** Type of sport practiced by the amateur athletes participating in the present study.

**Subject**	**Sport**	**Intensity per day**	**Times per week**	**Since (year ago)**
1	Endurance running	10 km	4	3
2	Endurance running	8 km	5	3
3	Weightlifting	2 h	5	3
4	Endurance running	10 km	7	3
5	Taekwondo	2 h	7	5
6	Soccer	10 km	4	6
7	Taekwondo	2 h	4	2
8	Endurance running	7 km	5	6

All subjects signed informed consent, and the protocol was approved by the Local ethics committee of Medical School, National Polytechnic Institute. The experiments were designed and conducted according to the Helsinki declaration.

Subjects were asked not to drink coffee or cola drinks during 24 h prior to the experiment because caffeine increases the excitability of the neuromuscular system (Walton et al., 2003). EMG recordings were carried out on both medial gastrocnemius muscles simultaneously, with subjects in a supine position. To avoid the cancelation of signals (Tucker et al., 2005), the electrodes were located above or below the innervation region (Childers, 2004; Sheverdin et al., 2009). Before recording, the skin around the popliteal fossa and the belly of the medial gastrocnemius muscle were cleaned with alcohol and gauze. After this procedure, the surface electrodes for stimulation and recording were carefully placed on the skin. The array of these two recording bipolar electrodes had the same inter-electrode distance as the stimulation electrodes (2.0 cm). The inter-electrode distance was defined as the center to center distance between the conductive area of two bipolar electrodes (according to SENIAM project; Surface ElectroMyoGraphy for the Non-Invasive Assessment of Muscles). The recording and stimulation electrodes were placed on the medial gastrocnemius according to Tucker et al. (2005). To guarantee a similar placement of the bipolar electrodes pre- and post-training we used a picture of the electrode positions and their slight adjustment to obtain H-waves with the lower intensity of stimulation (Hermens et al., 2000).

**Figure 1:**
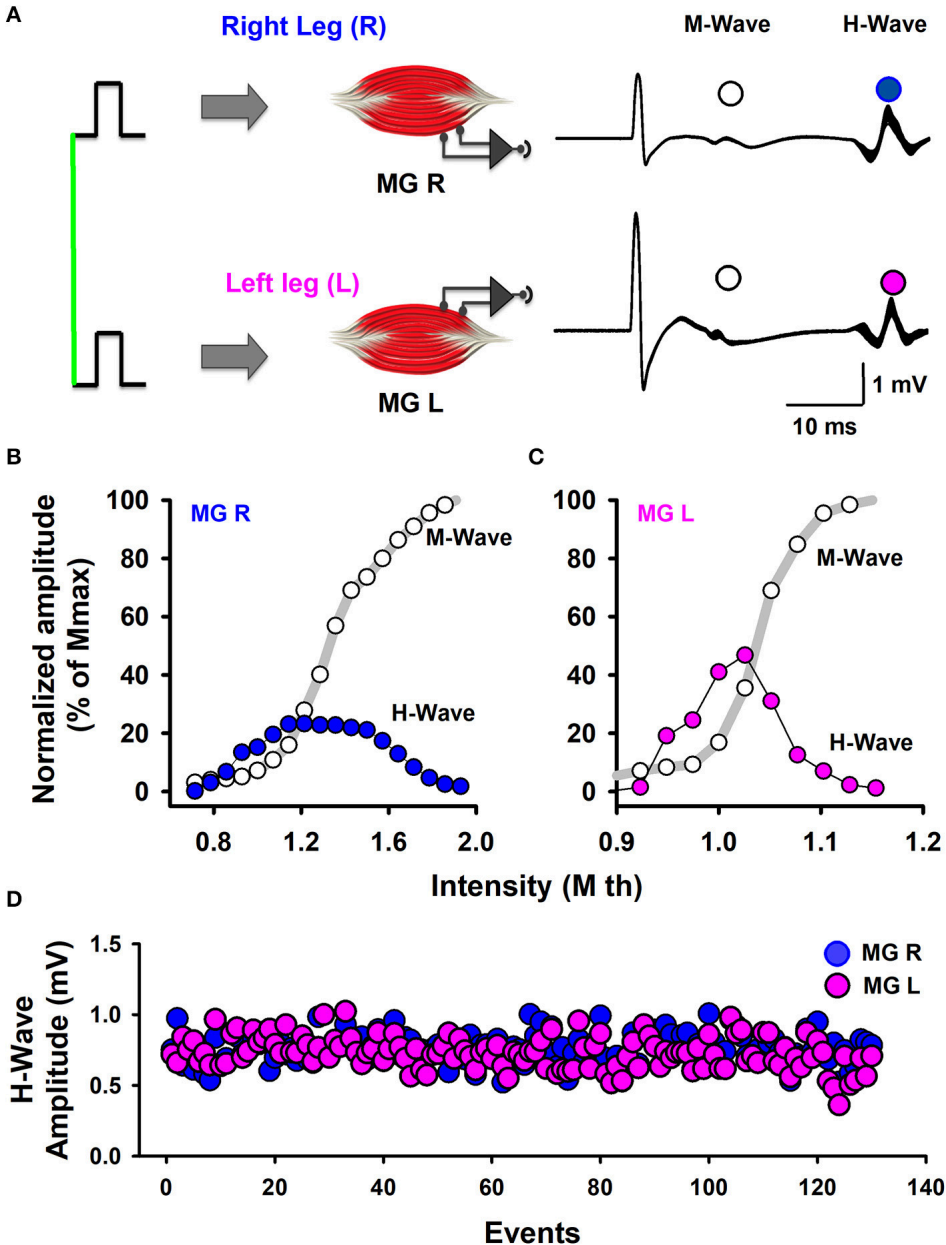
Scheme of the experimental arrangement. **(A)** Bilateral M-waves and H-waves were elicited by the simultaneous stimulation of Ia-afferents from the left and right legs. MGR is the right medial gastrocnemius muscle. MGL is the left medial gastrocnemius muscle. **(B,C)** Normalized amplitude of the M-waves and H-waves produced by the stimulation of both legs, as indicated. **(D)** The sequence of H-wave amplitudes from the left and right legs.

The stimuli were simultaneously applied to both legs with two independent stimulator units, using surface electrodes placed on the left and right popliteal fossa. The stimulation electrodes had an inter-electrode distance of 2.0 cm. The optimal stimulation site was the site in the medial gastrocnemius muscle in which we obtained H-waves with the lowest stimulation current. In all the experiments we performed slight adjustments in the position to obtain H-waves with the lower intensity of stimulation (Pierrot-Deseilligny and Mazevet, 2000). In this form, such stimuli activated the posterior tibial nerves and we obtained bilateral H-wave responses recorded in the right medial gastrocnemius (MGR) and left medial gastrocnemius (MGL) muscles (Figure 1A). First, recruitment curves were obtained (Figures 1B,C) for both muscles, and the stimulation intensities were adjusted to obtain bilateral H-waves with amplitudes of about 20% of the maximal M-wave amplitude (M_max_). This fact is clearly illustrated in our Figures 1B,C, which shows two H-wave recruitment curves, in which, it is clearly illustrated that at 20% of Mmax the H-wave amplitude is in the ascending part of the H-wave (see blue and magenta curves). Any reader could trace an imaginary horizontal line in 20% of Mmax in Figures 1B,C and could verify that the lower intensity to produce the H-wave at 20% of Mmax is in the ascending part of the recruitment curve. We used the intensity associated with the ascending part of the H-wave curve and not the descending part, which produces an antidromic collision. In this context, the use of 20% of Mmax in our experiments is in line with the recommendation to use H-wave lying in the ascending part of the recruitment curve (as described by Crone et al., 1990). Table 2 shows the range of intensities employed for the recruitment curves and the intensity at 20% of Mmax. Under these conditions, 130 bilateral H-waves were obtained (Figures 1B,C). Moreover, the time course of the peak to peak amplitude of the H-waves was measured, detrended and plotted (Figure 1D). Subsequently, the FD was computed using Higuchi's method.

**Table 2:** Range of stimulation intensities employed for the recruitment curves and the intensity at 20% of Mmax.

	**Range of intensities to obtain the recruitment curve for the left leg (mA)**		**Range of intensities to obtain the recruitment curve for the right leg (mA)**		**Intensity at 20% of Mmax to produce 130 H-waves for left leg (mA)**		**Intensity at 20% of Mmax to produce 130 H-waves for right leg (mA)**	
	**Before**	**After**	**Before**	**After**	**Before**	**After**	**Before**	**After**
**ATHLETES**								
1	5–25	6.6–11.95			7	6.8		
2	1.86–14	0.66–13.8	2.26–16	3.86–18	2.4	1.4	2.8	5.2
3	2.26–18.2	2.26–18.5	3.6–11.4	5.6–11.52	2.6	3	3.9	6.9
4			5.56–11.6	8.6–12.1			6.5	9.8
5	3.25–19.75	3.5–14.75	6–13.5	5.5–23.5	4.25	5.5	7.1	7
6	3.75–13	4.75–9.5			5	6.75		
7	3–10	4.5–18.5		8–22	4	6.75	9	10.5
8	5–13	4.6–9	4.4–10	5–11.5	6.4	6	6.25	6.2
**SEDENTARY**								
1	3.75–16.25	6.25–22.5	6–15	9–27	7.25	10	7.11	13.9
2	2.5–13.5	5–22.5	5–20	8.5–25.5	6.25	7.25	7.5	12.4
3			3.5–13				5.6	13.6
4			5–20	10–38			9	12.3
5	6.25–21.25	4.5–13.5	4–10.5	5.5–17.5	7.5	5.5	7.8	6
6	3–13	5–20	3.5–15	6–18	6	7.5	4.2	7.3
7	6.25–15.75	3.5–5.5	5.5–12.5	6–8.5	7.5	4.2	6.5	7
8		7.5–27.5		5.5–14	12.5	11.25	8.4	7.5

### Training program

The training modality in all subjects consisted of running three times per week (for 13 weeks) on a concrete road of 5 km. The subjects were monitored by a running coach. Table 3 shows the timeline for this training program. The intensity of the exercise was set according to the handbook by Macneill and The Sport Medicine Council of British Columbia (2012). In this book, the run plan was designed to turn anyone into a runner, without injury. Sports medicine physicians designed this training program. The subjects were instructed to run faster every week, and the tempo training was monitored. Table 4 describes the training duration in minutes (Min), the rating of perceived exertion scale (RPE) and the training load (TL) for athletes and sedentary subjects. The RPE is a reliable indicator to monitor exercise intensity. To obtain the RPE the subjects were instruted to report the subjective rate of the level of exertion during training. We used the RPE scale from 1 to 10. The number 1 was very light activity. The numbers 2–3 represented light activity. The numbers 4–5 indicated moderate activity. The numbers 6–7 represented vigorous activity. The numbers 8–9 very hard activity, and the number 10 represented maximal effort. To calculate the TL we multiplied the RPE by session duration in minutes. Table 4 shows the RPE and TL the first and the last day of the training program. Note that the TL evolved during the 13 weeks after training. The justification of this training program is that it is commonly employed for beginning runners to prevent injuries and to obtain health benefits.

**Table 3:** Timeline for the measurements (duration of the training, and pre- post-training).

**day 0 ->**
Pre-training
H-wave and M-wave recordings in resting conditions
**52 days ->**
Track training of 5 km, three times per week during 13 weeks
No recording
**day 53**
Post-training
H-wave and M-wave recordings in resting conditions

**Table 4:** Training load (TL) in athletes and sedentary subjects.

**Subjects**	**First day**			**Last day**			**Subjects**	**First day**			**Last day**		
**Athletes**	**Min**	**RPE**	**TL**	**Min**	**RPE**	**TL**	**Sedentary**	**Min**	**RPE**	**TL**	**Min**	**RPE**	**TL**
1	21	7	147	15	5	75	1	43	9	387	28	7	196
2	23	7	161	17	5	85	2	42	9	378	25	7	175
3	28	7	196	19	5	95	3	41	9	369	24	7	168
4	21	5	105	17	5	85	4	38	9	342	23	7	161
5	22	5	110	16	5	80	5	39	9	351	24	7	168
6	24	5	120	18	5	90	6	38	10	380	26	7	182
7	23	5	115	17	5	85	7	42	9	378	28	7	196
8	19	5	95	15	5	75	8	38	9	342	23	7	161
Mean	22.6	5.75	131	16.8	5	83.8		40.1	9.125	366	25.1	7	176
SD	2.67	1.04	34.2	1.39	0	6.94		2.1	0.354	18.2	2.03	0	14.2

*Min, minutes; RPE, rating of perceived exertion scale*.

### Higuchi's method

Higuchi's method is an algorithm for computing the fractal dimension in the time domain (Higuchi, 1988). We chose this method because it is suitable for calculating the FD during short time spans (Accardo et al., 1997). We only analyzed stationary series of H-waves. Recordings with movement artifacts were not considered for the analysis. We validated Higuchi's method with an analysis of the FD of the surrogate data obtained by a random shuffling of the original data (shuffled surrogates of the H-wave time series). Higuchi's method and these analyses were performed on MATLAB (MathWorks, Natick, MA, USA).

## Data acquisition system

### Stimuli

The stimulus to produce H-waves was applied simultaneously in both posterior tibial nerves to activate the MGR and MGL muscles (Figure 1A). Such stimuli consisted of constant current rectangular pulses, of 1 ms duration at 0.166 Hz (i.e., one pulse every 6 s). The current intensity was narrowed between 1.4 and 13.9 mA. The stimuli were generated with two independent stimulators (Digitimer, DS5 and DS7) commanded by another stimulator (Master-8), which also generated a trigger pulse to activate the data acquisition system (see the vertical green line, Figure 1A).

### H and M waves

The recorded electromyography signals, H-wave and M-wave (see superimposed recordings, Figure 1A, right panel), were amplified 500 times with two separate amplifiers (GRASS Astro-medic LP511), one for each leg, and filtered with a frequency range from 10 Hz to 1 kHz. The basal electrical activity of the muscles was monitored online with a digital oscilloscope (Tektronix TDS 2014). Finally, the signals were digitized with a Digidata 1440A interface (Axon CNS Molecular Device) having a sampling rate of 50 kHz, and stored for further analysis. Subsequently, the latencies and amplitudes (peak to peak) of the H-waves were measured with the AxoScope software version 10.2 (Molecular Devices, Silicon Valley, USA).

To avoid the depression transient due to “homosynaptic post-activation depression” for repetitive stimulation (see Hultborn et al., 1996) we used a very low stimulation frequency of 0.166 Hz. With this stimulation frequency each H-wave was produced every 6 s, and therefore we did not observe the post-activation depression (see Figure 1D). In other studies employing higher stimulation frequencies around 1 Hz (e.g., see Mezzarane and Kohn, 2002) the authors discarded from the analysis the first 10 H-waves to avoid in their analysis the depression transient due to the homosynaptic post-activation depression. In our case, it was not necessary to discard any H-wave because we employed a very low frequency of stimulation and we did not observe this phenomenon. We followed the recommendations made by Rossi-Durand et al. (1999), who employed stimulation time intervals higher than 3 s. This is consistent with studies by Nielsen et al. (1993) who employed stimulation time intervals of 4 s (i.e., 0.25 Hz).

### H-wave latency

We defined the H-wave latency as the time interval between the stimulus artifact and the first peak of the H-wave. We measured the H-wave latency for all the subjects before and after training. We used the H-wave latency as a criterion to detect a possible abnormality in the spinal function after training. With this index of measurement, we can examine whether the studied subjects suffered a lesion during the training program. Furthermore, because the H-wave latency is not as variable as the H-wave amplitude, we justify that it can be employed as a control to validate changes in the H-wave amplitude between two experimental conditions (before and after training).

### Recruitment curves

In a preliminary protocol, the intensity of the test stimuli was increased to obtain a recruitment curve (Funase et al., 1994; Hilgevoord et al., 1994). The stimulation intensities were increased until the disappearance of the H-wave by the collision of orthodromic and antidromic action potentials on efferent fibers. Such recruitment curves provide information about the excitability state of the spinal cord (Funase et al., 1996). The recruitment curves were also useful for determining the 100% activation of all the motor units (M_max_) in the region where the recording electrode was placed. For some subjects, it was not possible to obtain recruitment curves; therefore, these subjects were excluded from the study. Such subjects exhibited high amplitude M-waves and most of them were athletes with high muscle mass. Due to the collision, it was not possible to obtain the H-wave in these subjects. In fact, this has been a very common observation in our laboratory along several years of experience recording the H-wave.

**Figure 2:**
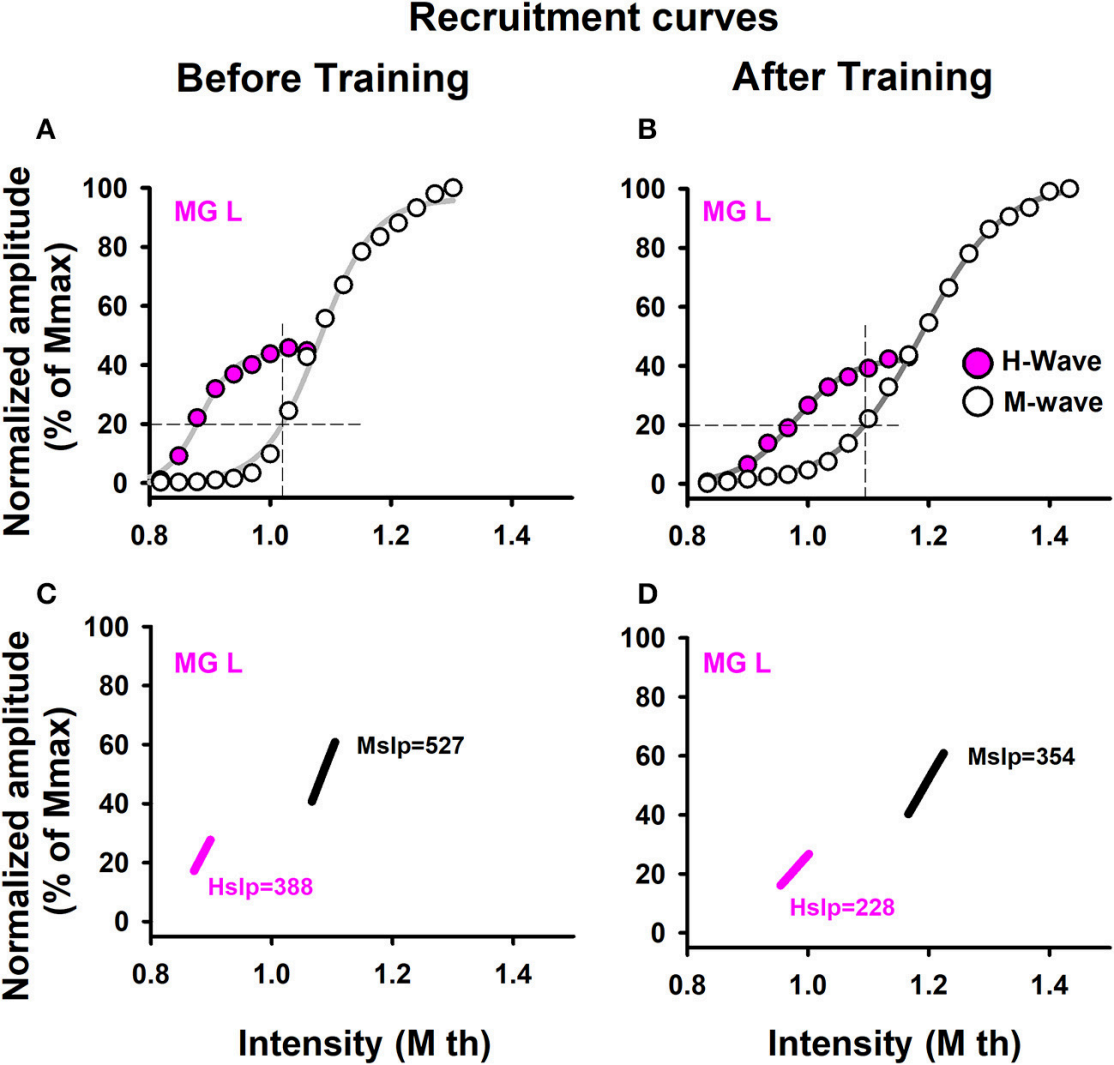
Recruitment curves. Data were obtained from one representative sedentary subject. Recruitment curves for the M-wave and H-wave: **(A)** before training and **(B)** after training. **(C,D)** Slopes for the M-wave and H-wave curves illustrated in **(A,B)**.

Data from the recruitment curves were fitted to sigmoid functions, and the slope of the ascending branches was computed (Klimstra and Zehr, 2008) (Figures 2C,D). We calculated the slope at 50% of the maximum H-wave amplitude (H_slp_), because this is the best way to obtain a fair value for spinal excitability (Klimstra and Zehr, 2008). We also computed the slope of the recruitment curves of the M waves (such slope were defined as: M_slp_). This method is shown for a sedentary subject (Figure 2). Moreover, we calculated the ratio H_max_/M_max_.

### Fractal dimension (FD) of H-wave amplitude fluctuations was computed with Higuchi's method

The FD was calculated with Higuchi's method with the algorithm written in MATLAB. The data employed to calculate the FD were the peak to peak amplitudes of the H-waves, as previously described. The analysis was performed from series of 130 H-waves, which were recorded during 13 min. Finally, a detailed statistical analysis was applied to examine whether there are significant differences between the mean FD obtained from different conditions.

### Cross-covariance

We computed the CCV of H-wave amplitude sequences recorded from both legs (simultaneous bilateral recordings). To compute the CCV we used a Matlab routine developed by Mezzarane and Kohn (2002). The time series of 130 H-wave amplitudes from both legs were detrended by subtraction of the best straight line fit to the time series. Such detrending was useful to avoid artifacts in the CCV analysis. We computed the 95% confidence interval using the formula 1.96/(N)^1/2^, where *N* = 130 (the number of samples in the H-wave sequence) (Brockwell and Davis, 1991; Mezzarane and Kohn, 2002).

### Statistical analysis

To test for any statistically significant difference in the H-wave and M-wave in sedentary individuals and athletes, we considered the maximum responses, slope and FD of M-wave and H-waves. Moreover, we tested for any statistically significant difference in the CCV of bilateral H-wave amplitude fluctuations. To compare the after vs. before training conditions for every item (M_max_, H_max_, M_slp_, H_slp_, H_max_/M_max_, FD, and CCV), we performed statistical analysis. Because some data are not normally distributed (Kolmogorov-Smirnov normality test, *p* < 0.05) and some have no homogeneity of variances (Levene test, *p* < 0.05), we used Friedman's ANOVA for non-parametric parameters. Where the differences were statistically significant, we performed the Wilcoxon signed-rank test with the null hypotheses that the difference between the aforementioned conditions was zero. The test of significance was one-tailed.

## Results

### Latency of H-wave in sedentary individuals

The mean latencies of the H-wave recorded in both MGR and MGL muscles in the athletes before and after training were 32.9 ± 0.4 and 33.1 ± 0.38 ms (mean ± standard error), respectively. The corresponding mean latencies of the H-waves in the sedentary individuals before and after training were 32.4 ± 1.5 and 32.7 ± 1.5 ms, respectively. The Student's *t*-test resulted in *t* = 1.34 and *p* = 0.2 for the athletes, while the values were *t* = 0.95 and *p* = 0.36 for the sedentary individuals. These data show that in both the athletes and sedentary individuals the H-wave latencies were not statistically modified after track training.

### Recruitment curves

To analyze recruitment curves we used two parameters: the maximal amplitudes of those waves (M_max_ and H_max_, which were measured from the curves, as illustrated in Figures 2A,B), as well as the slope of the ascending branch in the recruitment curves of the M and H waves (H_slp_ and M_slp_). The results are shown for the H_slp_ and M_slp_ obtained from a sedentary subject before and after training (Figures 2C,D). The slopes for the M-wave recruitment curves are illustrated in magenta, and the slopes for the respective H-wave recruitment curves in black (Figures 2C,D). Similar graphs were obtained for all the subjects (both the athletes and sedentary individuals) and the data were collected for their statistical analysis. Table 5 shows raw data per subject, for athletes and sedentary subjects. Such table shows the data in mV for the left and right legs, for H-waves, M-waves and H_max_/M_max_ ratio before and after training.

**Table 5:** Summary of raw data per subject for Hmax, Mmax, and their ratio.

	**Before (right)**		**H/M**	**Before (left)**		**H/M**	**After (right)**		**H/M**	**After (left)**		**H/M**
	**Mmax (mV)**	**Hmax (mV)**		**Mmax (mV)**	**Hmax (mV)**		**Mmax (mV)**	**Hmax (mV)**		**Mmax (mV)**	**Hmax (mV)**	
**ATHLETES**												
1	2.22	1.24	0.56	2.24	0.98	0.44	1.71	0.75	0.44	2.24	0.46	0.21
2	2.72	1.44	0.53	1.85	1.22	0.66	5.61	1.43	0.26	2.57	0.95	0.37
3	2.08	0.43	0.21									
4	1.12	0.31	0.28				3.78	1.36	0.36	3.89	1.77	0.45
5	3.35	1.06	0.32	3.13	1.10	0.35	4.96	1.33	0.27	4.49	1.48	0.33
6	1.22	0.58	0.48	1.59	1.31	0.83	2.11	1.14	0.54	2.94	1.04	0.36
7	2.43	1.50	0.62	1.73	0.96	0.56	1.76	0.58	0.33	3.13	0.75	0.24
8							3.21	1.17	0.37	3.04	1.07	0.35
Mean	2.16	0.94	**0.43**	2.11	1.11	**0.57**	3.31	1.11	**0.37**	3.19	1.07	**0.33**
SD	0.79	0.49	**0.16**	0.62	0.15	**0.19**	1.56	0.32	**0.10**	0.77	0.43	**0.08**
**SEDENTARY**												
1	2.86	1.09	0.38	1.14	0.93	0.82	2.17	1.19	0.55	1.86	1.22	0.66
2	4.10	0.59	0.14				4.00	0.82	0.21	3.34	0.35	0.11
3							2.53	1.74	0.69	2.54	1.86	0.73
4							4.01	1.48	0.37	2.67	1.63	0.61
5							1.66	1.47	0.88	2.63	0.97	0.37
6	4.93	1.35	0.27	2.67	1.21	0.45	5.10	1.17	0.23	3.32	1.15	0.35
7	2.98	1.63	0.55	4.83	1.75	0.36				3.13	0.86	0.27
8	3.66	0.35	0.10	3.40	0.67	0.20	5.23	0.41	0.08	1.21	0.55	0.46
Mean	3.70	1.00	**0.29**	3.01	1.14	**0.46**	3.53	1.18	**0.43**	2.59	1.07	**0.44**
SD	0.85	0.52	**0.18**	1.53	0.46	**0.26**	1.42	0.44	**0.29**	0.74	0.50	**0.21**

*The values in bold character are the mean and standard deviation of the left and right limbs for sedentary and athlete subjects*.

To examine the statistical significance of the comparison of mean values for M_max_ and H_max_ (and H_max_/M_max_) in sedentary individuals and athletes before and after training, we performed two nonparametric Friedman tests among the four conditions (M_max_ before, M_max_ after, H_max_ before and H_max_ after) for all subjects. For sedentary individuals, the results show highly significant differences between the four conditions [$\chi_{\left(3\right)}^{2}$ = 25.15, *p* = 0.00001]. A subsequent *post hoc* analysis using the Wilcoxon sign test revealed significant differences between M_max_ before and M_max_ after (*p* = 0.008) (see black arrow in Table 6), but not between H_max_ before and H_max_ after (*p* = 0.361). Similarly, the data for athletes showed significant differences between the four conditions [$\chi_{\left(3\right)}^{2}$ = 16.96, *p* = 0.001]. However, the *post hoc* analysis using the Wilcoxon sign test revealed no significant difference between M_max_ before and M_max_ after (*p* = 0.779), nor between H_max_ before and H_max_ after (*p* = 0.99) (see Table 6).

**Table 6:** Results obtained from the statistical analysis.

**Variables**	**Friedman's ANOVA**				**Wilcoxon signed-rank test (p)**		
	***n***	**Chi2**	***df***	***P***	**Before vs. After**		
**ATHLETES**							
M_max_	8	16.96	3	0.001	0.779		ns
H_max_	8	16.96	3	0.001	0.99		ns
M_slp_	13	14.87	3	0.091	–		ns
H_slp_	13	14.87	3	0.091	–		ns
H_max_/ M_max_	6	5.00	3	0.172	–		ns
FD	13	19.46	3	0.0002	0.001		^*^ 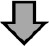
CCV	11	9	3	0.029	0.02		^*^ 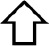
**SEDENTARY**							
M_max_	11	25.15	3	0.00001	0.008		^*^ 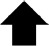
H_max_	11	25.15	3	0.00001	0.361		ns
M_slp_	13	2.52	3	0.472	–		ns
H_slp_	13	2.52	3	0.472	–		ns
H_max_/ M_max_	6	5.00	3	0.172	–		ns
FD	14	19.46	3	0.0002	0.006		^*^ 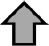
CCV	11	9	3	0.029	0.08		ns 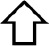
**Athletes vs. sedentary individuals**							
**Variables**	***n***	**Chi2**	***df***	***P***	**Before vs. Before**	**After vs. After**	
FD	13	19.46	3	0.0002	0.001	0.391	
CCV	11	9	3	0.029	0.225	0.08	

*Because some of the data were not normally distributed (Kolmogorov-Smirnov normality test, p < 0.05) and some of them had no homogeneity of variances (Levene test, p < 0.05) we used a non-parametric Friedman's ANOVA. Where the differences were statistically significant, we performed the Wilcoxon signed-rank test with the null hypotheses that the differences between the aforementioned conditions were zero. The test of significance was one-tailed. P-values are shown for the Friedman's ANOVA and the subsequent Wilcoxon signed-rank test. These p-values indicate the degree of significance in the difference between values before and after training for M_max_, H_max_, M_slp_, H_max_/M_max_, H_slp_, FD, and CCV (Before vs. After). Values of p < 0.05 were considered significant for the Wilcoxon signed-rank test, and they are indicated with an asterisk and arrow. The direction of the arrows indicates the direction of the change (i.e., an arrow going up indicates an increase in the mean value, and vice versa). The gray arrows are for the change in FD and the white arrows for the change in CCV. The black arrow illustrates the change found in the M_max_ for sedentary individuals after training. The mean values are described in the main text. Ns, statistically non-significant*.

Likewise, we examined the statistical significance of the comparison of mean values of the slopes, M_slp_ and H_slp_, for sedentary individuals and athletes before and after training. We performed two nonparametric Friedman tests between the four conditions (M_slp_ before, M_slp_ after, H_slp_ before and H_slp_ after) for all subjects. The data analysis showed no significant differences between the four conditions [$\chi_{\left(3\right)}^{2}$ = 2.52, *p* = 0.472 for sedentary individuals, and $\chi_{\left(3\right)}^{2}$ = 14.87, *p* = 0.091 for athletes; see Table 6].

Moreover, we examined the statistical significance of the comparison of Hmax/Mmax in sedentary and athletes, before and after training (see Table 5). We performed a nonparametric Friedman test to the data obtained from the four conditions: “sedentary before,” “sedentary after,” “athletes before,” and “athletes after.” The data analysis showed no significant differences among the four conditions [χ2_(3)_ = 5, *p* = 0.172]. See Table 6.

Overall, the results strongly suggest that training did not produce changes in spinal excitability related to the gain in amplitude of the recorded H-waves in either group of subjects.

### Return maps

**Figure 3:**
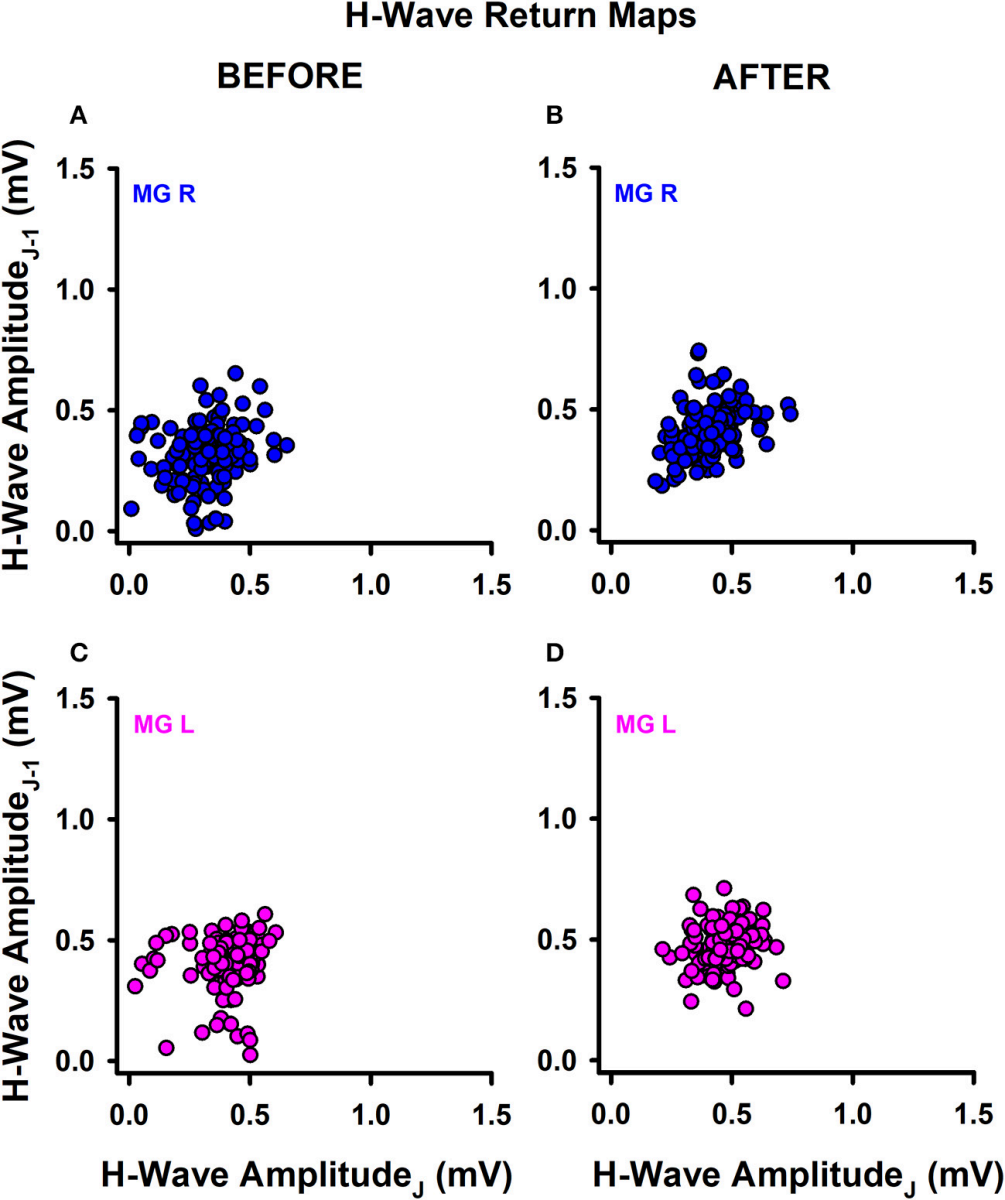
Return maps obtained from successive H-waves. **(A)** MGR amplitude of H-waves before training. **(B)** MGR amplitude of H-waves after training. **(C,D)** The same as **(A,B)** but for the MGL.

We also explored the qualitative behavior of H-wave amplitude fluctuations for sedentary individuals and athletes before and after training. Return maps were constructed for the incoming peak-to-peak H-wave amplitude on the abscissa (j) vs. the previous peak to peak H-wave amplitude (j-1) on the ordinate (example shown in Figure 3 for one athlete).

Data were recorded on the right leg (Figures 3A,B) and left leg (Figures 3C,D), before training (Figures 3A,C) and after training (Figures 3B,D). In general, we observed a mild change in the data dispersion after training in both groups of subjects, without significant changes in the mean plus standard deviation for any subjects or conditions. This qualitative finding led us to examine whether there are changes in other measurements obtained from the data before and after training, such as the fractal dimension (FD) of the H-wave amplitude fluctuations illustrated in the return maps.

### Fractal dimension (FD)

The return maps were not sufficient for detecting changes in the behavior of amplitude fluctuations of the H-wave. Therefore, we computed the FD of such amplitude H-wave fluctuations for all subjects before and after training. The main idea behind the FD value is that it represents a relative index of complexity. Therefore, we employed this index to detect changes in the complexity of the H-wave amplitude fluctuations before and after track training.

**Figure 4:**
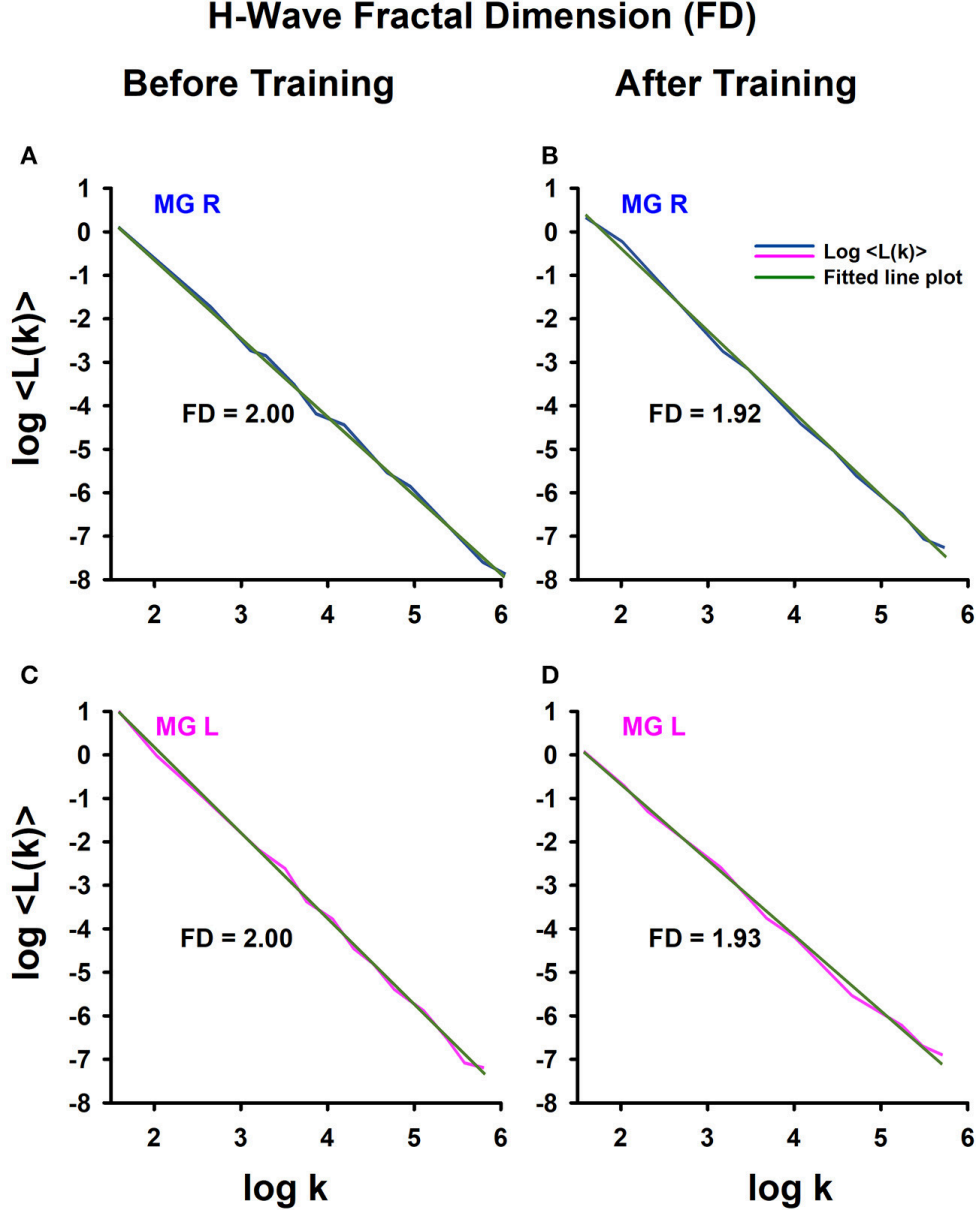
H-wave fractal dimension (FD) computed with Higuchi's method. **(A)** The FD was calculated from data: **(A)** before training and **(B)** after training. The MGL muscle responses are from: **(C)** before training and **(D)** after training.

To illustrate how we obtained the FD, an example is shown of the FD analysis for one athlete (Figure 4), obtaining data from the right leg (Figures 4A,B) and left leg (Figures 4C,D), before training (Figures 4A,C) and after training (Figures 4B,D). Note that for this athlete, the FD in the amplitude fluctuations of the right H-wave changed from 2 to 1.92 (before vs. after track training), and similarly the FD for the amplitude fluctuations of the left H-wave changed from 2 to 1.93. In general, we systematically observed this type of change in the FD for all the athletes.

Graphs similar to the one illustrated in Figure 4 were obtained for all subjects (athletes and sedentary individuals). The data were collected for statistical analysis.

We employed the nonparametric Friedman test to examine the statistical significance of the FD measurements for the four conditions: sedentary individuals before, sedentary individuals after, athletes before and athletes after. The analysis of data showed significant differences between the four conditions [$\chi_{\left(3\right)}^{2}$ = 19.45, *p* < 0.0002]. A subsequent *post hoc* Wilcoxon sign test revealed significant differences between sedentary individuals before and after training (*p* = 0.006), athletes before and after training (*p* = 0.001), sedentary individuals before and athletes before training (*p* = 0.001), but not between sedentary individuals after and athletes after training (*p* = 0.391). It is important to point out that although the absolute value of the FD between sedentary individuals and athletes was similar after training, the direction of the change in the FD was opposite between the two groups of subjects. Whereas the FD index significantly increased for the sedentary individuals, from 1.88 ± 0.12 to 1.94 ± 0.05 (*p* = 0.006, *n* = 14), this parameter significantly decreased for the athletes, from 1.98 ± 0.01 to 1.93 ± 0.04 (*p* = 0.001, *n* = 13) (see Table 6).

### Cross-covariance (CCV)

**Figure 5:**
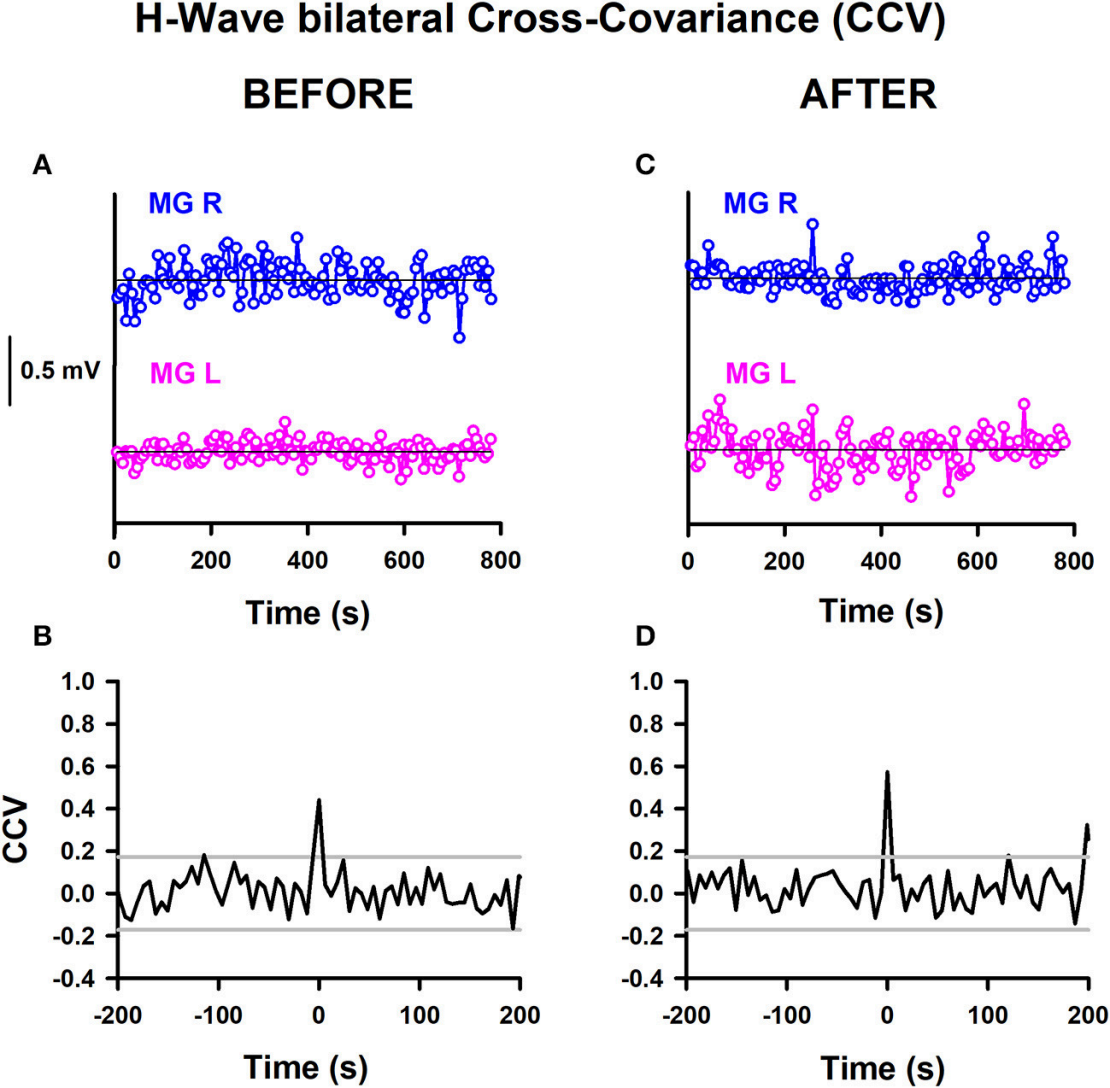
H-wave bilateral cross-variance before and after training. **(A)** Sequence of H-wave amplitude fluctuations for the MGR and MGL: **(A)** before training and **(C)** after training. CCV of H-wave amplitude fluctuations: **(B)** before training and **(D)** after training. The data obtained from both legs were detrended by subtraction of the best straight line fit to the time series.

We calculated the CCV from time series of bilateral H-wave amplitude fluctuations. This could be useful for examining whether there are common synaptic inputs modulating the spinal alpha-motoneuron pools from the two legs. A significant change in the CCV amplitude at a particular lag would indicate a stronger modulation of the alpha-motoneuron pools from both legs or a stronger modulation from common central inputs on the alpha-motoneurons. We computed such CCVs before and after training in the two groups of subjects, athletes and sedentary individuals. Therefore, the aim of this analysis was to examine whether the CCV could be useful as a biomarker to detect plastic changes produced by track training in healthy subjects. The CCV analysis is shown for one athlete before and after training (Figure 5). Similar analyses were performed for all the subjects. The detrended time series of the H-wave amplitudes for the right (MGR) and left (MGL) legs is represented (Figure 5A). The horizontal lines illustrate the corresponding reference baselines associated with the detrending. The corresponding CCV obtained from such time series is presented (Figure 5B). The two horizontal lines indicate the 95% confidence interval. A clear CCV peak at lag 0 emerges above the confidence band (Figure 5B). We observed that this CCV exhibited a significant increase for athletes (comparing such CCV peaks in Figures 5B,D). Similar results were obtained for all the CCVs computed for athletes. In sedentary individuals, contrarily, we did not observe a significant change in the CCV peak after track training.

The mean amplitude of the CCV peak at lag 0 for the athletes before training was 0.21 ± 0.19, changing to 0.47 ± 0.13 after training. Meanwhile, the mean amplitude of the CCV peak at lag 0 for the sedentary individuals before training was 0.08 ± 0.12, changing to 0.24 ± 0.21 after training. We employed the nonparametric Friedman test to examine the statistical significance of the CCV between the bilateral H-wave amplitudes for the distinct conditions: athletes before, athletes after, sedentary individuals before and sedentary individuals after. The results show significant differences between the four conditions [$\chi_{\left(3\right)}^{2}$ = 9, *p* < 0.029]. In the CCV, the Wilcoxon sign test revealed a significant difference between athletes before and after (*p* = 0.02). In contrast, no statistically significant difference was found between sedentary individuals before and after (*p* = 0.08), sedentary individuals before and athletes before (*p* = 0.225), or sedentary individuals after and athletes after (*p* = 0.08) (see Table 6).

### Summary of results for the FD and the CCV

**Figure 6:**
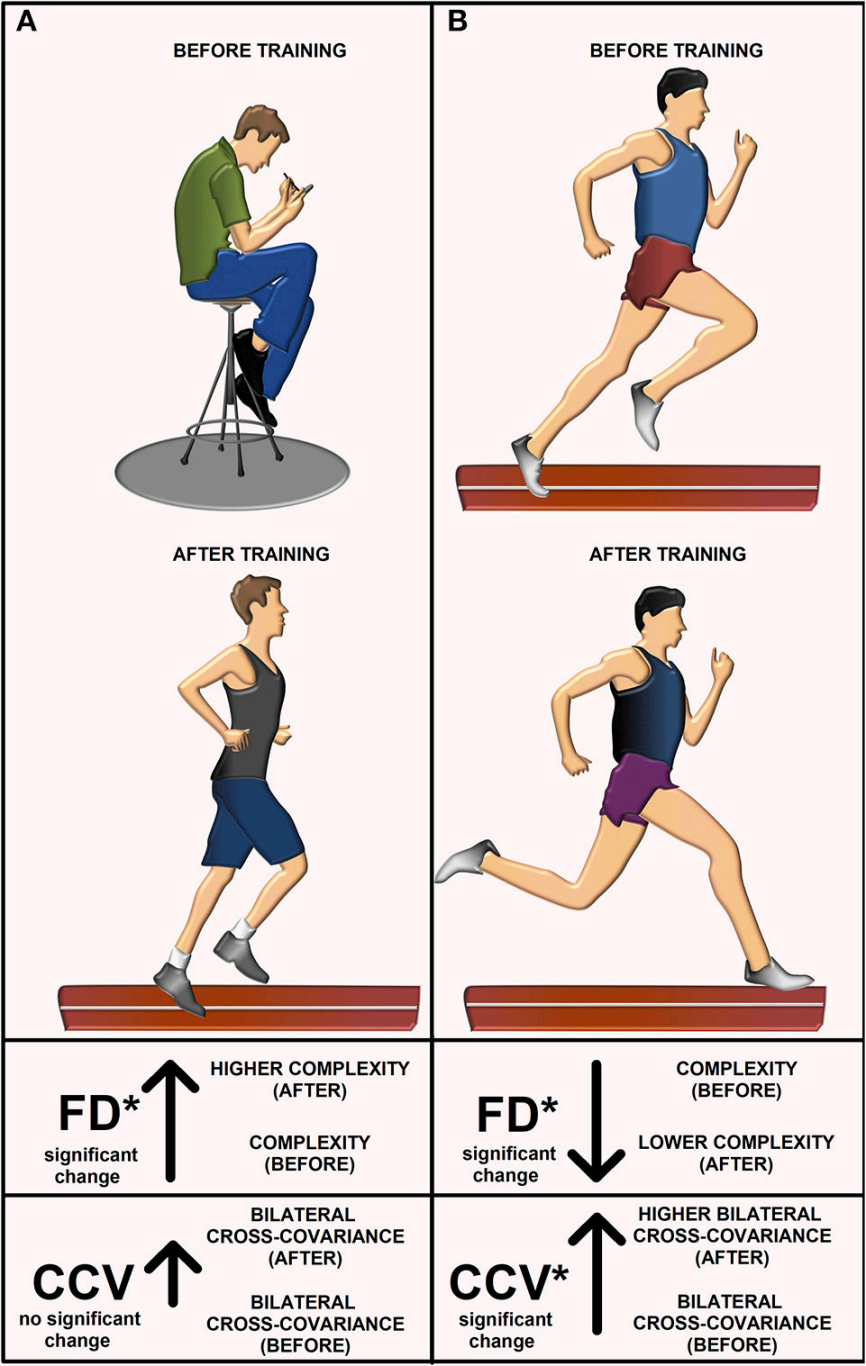
The cartoon explains the main results. Illustration before and after training for **(A)** sedentary individuals and **(B)** athletes. The FD for sedentary individuals increased after track training. They exhibited a non-significant increase in the cross-covariance CCV between left and right H-wave fluctuations. The FD for athletes decreased and the CCV increased.

We herein highlight the statistically significant changes for the FD and CCV obtained for athletes and sedentary individuals before and after track training. In Table 6 (last column), we point out such significant changes with gray and white arrows and the illustrated asterisks. Note that the FD for the athletes goes down (gray arrow). Conversely, the FD for the sedentary individuals goes up (gray arrow). In both cases, the changes are statistically significant (see asterisk). The conclusions from this statistical analysis for athletes and sedentary individuals before and after track training is illustrated with a cartoon (Figure 6). The CCV for both the athletes and sedentary individuals goes up (white arrows, Table 6, last column). However, only the athletes exhibited a statistically significant increase in the CCV, whereas for the sedentary individuals this increase did not reach statistical significance. The change in the CCV of the bilateral H-waves for the athletes and sedentary individuals is also presented (Figure 6).

## Discussion

We found that after a track training program, the FD of the H-wave amplitude fluctuations significantly increased in sedentary individuals but decreased in athletes. Furthermore, we found that only the athletes exhibited a statistically significant increase in the CCV after training, whereas for the sedentary individuals this increase did not reach statistical significance. These differential findings suggest that the FD and CCV are dependent on the previous fitness history of the subjects.

The rationale behind the use of FD and CCV to understand neural plasticity following training is that both indexes are associated with the H-wave amplitude fluctuations. These types of analyses add a new form to study plastic changes in the H-wave pathways. They are justified because most of the analyses of plasticity in the H-wave circuits only involve the H-wave amplitude.

To understand the rationale behind the use of FD and CCV for the H-wave fluctuations is important first to describe with some detail the accepted basic principles by which the H-wave is produced. Briefly, an H-wave can be elicited if electrical stimulation of the nerve is above the threshold for activation of Ia afferents and the afferent terminals can release neurotransmitter at the Ia afferent to the alpha-motoneuron synapse. If the postsynaptic potentials reach the threshold, then the alpha-motoneurons will produce action potentials, which in turn, will produce a dramatic depolarization and contraction of muscle fibers, which finally will be recorded as the H-wave. The amplitude of the H-wave is due in part to the recruitment of motor units, which occurs in an orderly fashion, from smallest to largest, following the “Henneman size principle” (Henneman et al., 1965; Somjen et al., 1965). Such recruitment could be elicited by activation of corticospinal or Ia afferent inputs. However, it is important to mention that the H-wave is not a direct measure of alpha-motoneuron excitability due to the effect of presynaptic inhibition on the monosynaptic reflex pathway. This presynaptic inhibition from other afferent inputs considerably affects the H-wave amplitude (Stein, 1995; Pierrot-Deseilligny, 1997), the variability (Rudomin and Dutton, 1967), as well as the local control of information flow in segmental and ascending collaterals of single afferents (Lomelí et al., 1998). Other physiological factors could also affect the amplitude of the H-wave, as the alpha-motoneuron intrinsic properties (Hounsgaard, 2017) or the background electrical activity of the motoneurons produced by synaptic inputs from other spontaneously active spinal neurons (Manjarrez et al., 2000, 2005). In this context, there are diverse physiological factors that could produce changes in the H-wave amplitude. Therefore, any measure involving the H-wave amplitude fluctuations may be associated with the physiological mechanisms above mentioned. Such is the case of the H-wave FD, which is a measure of the complexity level of the H-wave amplitude fluctuations (Nozaki et al., 1995). Any change in the H-wave FD could be attributed to a change in the complexity of neurotransmitter release, alpha-motoneurons recruitment, presynaptic inhibition, or intrinsic properties of alpha-motoneurons, among other factors. Our result that the H-wave FD in sedentary subjects increased after training, and conversely, it decreased in athletes after training (Figure 6), suggest that the training modified some of the abovementioned physiological mechanisms related to the H-wave fluctuations in a differential fashion. Future studies will be necessary to examine specific mechanisms producing this differential change in the H-wave FD. Such studies will be challenging because the physiological mechanisms behind the FD in neural systems are unknown. The more intuitive reports about the FD are related to the fractal morphometry of cell complexity (Losa, 2002, 2009). However, other reports, in particular in the neurosciences do not explain the physiological mechanisms behind the FD and they only define the FD as a measure of complexity (Cross, 1997; Di Ieva et al., 2014, 2015; Kesić and Spasić, 2016).

The finding that sedentary subjects exhibited an increase of the H-wave FD after training is intuitive. Before training, the sedentary subjects may exhibit H-wave fluctuations with low complexity given the limited use of their monosynaptic reflex pathway, due to the low physical activity. Therefore, after training the high physical activity of these sedentary subjects may produce a complex change in the alpha-motoneurons (Gardiner et al., 2006), or in other spinal neural elements, which could be reflected as a higher H-wave FD. However, although intuitive, this possibility must be taken with caution because numerous neuronal elements in the brain could also contribute to increasing such H-wave FD. Conversely, as illustrated in Figure 6, we found that the athletes exhibited a counterintuitive decrease of the H-wave FD after training. The description of possible mechanisms behind this finding is more challenging and also more difficult to understand, given the great number of factors mediating the H-wave fluctuations, as explained above. Just we could mention that the athletes also exhibited a concomitant increase in the CCV of the bilateral H-wave fluctuations after training (i.e., more similarity between left and right H-waves), and this could reflect more organization in their sequence of H-wave fluctuations. Such higher organization in athletes is consistent with the finding of a lower complexity of their H-wave fluctuations, and therefore, this could explain their lower H-wave FD after training. However, further studies will be necessary to understand the precise mechanisms involved in these findings for both athletes and sedentary subjects.

Other rationale behind the use of FD and CCV to understand the neural plasticity after training is that both are indexes that can be employed to analyze in more detail the H-wave fluctuations. Of course the variance is a good statistics to measure such fluctuations, however, the FD account for a measurement of the complexity of H-wave fluctuations, and the CCV for a measure of the magnitude of similarity between the amplitude fluctuations of bilateral H-waves.

In several situations of training, as track running and volleyball, the H-wave amplitude and the Hmax/Mmax ratio are lower in athletes compared to control subjects (Rochcongar et al., 1979; Casabona et al., 1990; Ozmerdivenli et al., 2002). However, in other types of training the Hmax/Mmax ratio is higher in trained subjects compared to ballet dancers (Nielsen et al., 1993). This discrepancy could be due to the type of motor task (i.e., aerobic vs. anaerobic). In general, Rochcongar et al. (1979) described that the Hmax/Mmax ratio is higher for subjects involved in aerobic exercise and lower for subjects performing anaerobic training. Therefore, these differences suggest that the H-wave amplitude and Hmax/Mmax ratio are good indexes for synaptic plasticity. Here we show that in addition to these indexes we can employ the FD and CCV of the H-wave fluctuations as new indexes to examine changes in synaptic plasticity after training. The physiological mechanisms associated with changes in the FD and CCV of H-wave fluctuations could be the same as the physiological mechanisms for changes in the H-wave amplitude and Hmax/Mmax ratio. Some of these mechanisms have been suggested in previous studies, and although they are highly speculative, they provide new research lines. For example, an increase in the presynaptic inhibition of the monosynaptic reflex pathway could explain the reduction of the H-wave in ballet dancers (Nielsen et al., 1993). A similar mechanistic explanation could be given for our findings. It is possible that the differential changes in the FD and CCV observed in athletes and sedentary subjects after training could be due to changes in presynaptic inhibition of the monosynaptic reflex pathway.

Our results show that even that no significant changes occurred in the H-wave amplitude and Hmax/Mmax there are clear changes in the FD and CCV of the H-wave fluctuations. This indicates that the H-wave fluctuations also convey important information associated with the motor system. This is in line with classical studies by Rudomin and Dutton (1969), in which it was demonstrated that a reduction in the variance of the monosynaptic reflex fluctuations is related to a presynaptic-inhibition mechanism even though the mean monosynaptic-reflex amplitude did not change.

The finding that the H-wave amplitude did not change after training in athletes and sedentary subjects was surprising. It could be due to the training modality that was used and especially the training intensity. The training modality in all subjects consisted of running three times per week (for 13 weeks) in a concrete road of 5 km. The justification to employ this training modality was that it was not too demanding for sedentary subjects and not too simple for athletes. Similar training modalities are commonly employed for beginning runners to prevent injuries and to obtain health benefits (e.g., see handbook by Macneill and The Sport Medicine Council of British Columbia, 2012). The type of athletes (amateurs) involved in the present study was diverse, and they practiced endurance running, weightlifting, taekwondo or soccer (see Table 1). This diversity of athletes and sedentary subjects could explain why we did not observe a significant change in the H-wave amplitude of such group of subjects after training. In future studies will be interesting to select only endurance running athletes and to examine whether all the three variables H-wave amplitude, FD and CCV, change in this group. Based on our result that the FD and CCV in athletes changed after training, but not the H-wave amplitude, we suggest the occurrence of only a partial change in motor performance in these subjects after training.

The finding that sedentary subjects exhibited an increase of the H-wave FD after training is intuitive. Before training, the sedentary subjects may exhibit H-wave fluctuations with low complexity given the limited use of their monosynaptic reflex pathway, due to the low physical activity. Therefore, after training the high physical activity of these sedentary subjects may produce a complex change in the alpha-motoneurons (Gardiner et al., 2006), or in other spinal neural elements, which could be reflected as a higher H-wave FD. However, although intuitive, this possibility must be taken with caution because numerous neuronal elements in the brain could also contribute to increasing such H-wave FD. Conversely, as illustrated in Figure 6, we found that the athletes exhibited a counterintuitive decrease of the H-wave FD after training. The description of possible mechanisms behind this finding is more challenging and also more difficult to understand, given the great number of factors mediating the H-wave fluctuations, as explained above. Just we could mention that the athletes also exhibited a concomitant increase in the CCV of the bilateral H-wave fluctuations after training (i.e., more similarity between left and right H-waves), and this could reflect more organization in their sequence of H-wave fluctuations. Such higher organization in athletes is consistent with the finding of a lower complexity of their H-wave fluctuations, and therefore, this could explain their lower H-wave FD after training. However, further studies will be necessary to understand the precise mechanisms involved in these findings for both athletes and sedentary subjects.

The analysis of CCV between bilateral H-waves merits a discussion whether there is interference between the spinal responses elicited by both stimuli. To our knowledge, there is no such interference between both H-waves in humans (Mezzarane and Kohn, 2002). There are some studies in animals indicating that the contralateral stimulation of group I afferents present a weak direct influence upon the ipsilateral motor nucleus (Harrison and Zytnicki, 1984). Consistently, there is evidence that the stimulation group Ia afferents did not produce contralateral monosynaptic reflexes in cats (Manjarrez et al., 2005) or humans (Mezzarane and Kohn, 2002). Moreover, in a subsequent study from our laboratory, we demonstrated an absence of contralateral group I muscle afferents on presynaptic inhibition of Ia terminals in humans and cats (Mezzarane et al., 2012).

Mezzarane and Kohn (2002), also applied bilateral stimulation on both legs as in the present study. According to Mezzarane and Kohn (2002), the main idea of this type of simultaneous bilateral stimulation was to use the CCV to examine whether there are correlated or common inputs modulating the motoneuron pools, interneurons and associated Ia terminals of the muscles from the two legs. In our study, we followed a similar stimulation protocol as Mezzarane and Kohn (2002) to examine whether the training could produce differential changes in the CCV in sedentary and athletes. Our results show that such differential changes in the CCV occur in athletes (the CCV was increased after training) but not in sedentary subjects. Further studies will be necessary to understand the physiological mechanism behind this difference.

### Plasticity

We decided to perform the recordings in the gastrocnemius muscle instead of the soleus muscle because it is composed of approximately 50% of rapid and 50% of slow muscle fibers. However, the soleus muscle is composed by 70–90% of slow muscle fibers and only 10–30% of rapid muscle fibers (Johnson et al., 1973; Edgerton et al., 1975; Green et al., 1981). Given the type of track training in our study, subjects running only during 13 weeks, we assumed that plastic changes could be due to rapid muscle fibers.

Plasticity in the spinal cord is a mechanism of the nervous system useful to resolve challenges in motor behavior as well as in learning new motor skills. In this context, we observed that in athletes the TL before and after training changed from 131 ± 34.2 to 83.8 ± 6.94 and in sedentary subjects from 366 ± 18.2 to 176 ± 14.2. These results suggest that the training program employed in these subjects improved the motor performance in both athletes and sedentary subjects, thus suggesting that plastic changes could occur after track training. It is known that with the voluntary training, the neurons show evidence of dendritic grow, increase in the synthesis of new proteins, increase in protein axonal transport and changes in the intrinsic properties of motoneurons (see revision in Gardiner et al., 2006). It is now widely accepted that the H-wave is a tool for studying such plastic changes in the spinal cord (Wolpaw and Tennissen, 2001; Zehr, 2002). Indeed, skills are adaptive behaviors that are acquired through practice and plastic changes in the spinal cord (Grillner and Wallen, 2004; Chen et al., 2005). Moreover, the spinal cord is the first place where plastic changes associated with motor behavior can occur, since this structure has a large quantity of switching circuits susceptible to neuronal synaptic efficacy (Wolpaw and Tennissen, 2001). Changes in the synaptic transmission efficacy may result in plastic events in the same synapse, or in other synapses throughout the selective presynaptic control (Quevedo et al., 1997; Lomelí et al., 1998). In this context, it would be interesting to examine whether the selective presynaptic control could also change after training in our experimental paradigm.

Changes in the synaptic plasticity of the monosynaptic reflex pathway occur not only after training but also after spinal cord injury. Lee et al. (2009) demonstrated that a better functional outcome of compression spinal cord injury in mice is related to alterations of the monosynaptic reflex pathway which facilitate mononeuron recruitment. These authors showed that such facilitation occurred spontaneously after the spinal cord lesion and it was associated with an increase of the H-wave amplitude 6–12 weeks after the injury. In our case we observed a differential change in the FD and CCV of the H-wave fluctuations without significant changes in the H-wave amplitude after training in both athletes and sedentary subjects. A possible explanation for this discrepancy could be that the plastic changes that we observed could occur at the level of mechanisms controlling the H-wave fluctuations via presynaptic actions and not exclusively at the level of the Ia-motoneuron synapse.

In future experiments, it would be interesting to explore the effects of diverse substances that affect H-wave fluctuations, including caffeine, which produces profound alterations in neural function through known mechanisms (Rivera-Oliver and Diaz-Rios, 2014). The possibility of studying the FD and CCV changes in this context could be of interest for physiologists and physicians. However, caution must be taken because it is difficult to establish cause-effect associations in this emergent field known as the neuroscience of exercise (Machado et al., 2015). We conclude that the observed changes in the CCV between bilateral H-waves, as well as the differential changes in the FD of the H-wave amplitude fluctuations, are associated with the previous fitness history of the subjects. Further studies on animals and humans are necessary to describe the physiological mechanisms underlying such associations in detail (see also Reyes et al., 2007).

### Functional implications

Previously, it was demonstrated in cats that the intraspinal branches of afferent fibers function as a dynamic assembly that can be fractionated to convey information to selected neuronal targets in the different spinal nucleus (Lomelí et al., 1998) or inside of the same neuronal nucleus (Quevedo et al., 1997). Therefore, it is plausible that in humans, the selective presynaptic action of such afferent fiber collaterals on different neuronal targets could also contribute to changes in the FD index and the CCV after a track training program. In future experiments, it would be interesting to examine whether the strength of presynaptic actions of afferent fiber collaterals and their selectivity on diverse spinal neuronal groups also change in cats after a track training program.

In the present experiments, the intensity of stimulation was very low for the activation of group II fibers. However, because the electrical threshold for activating Ia afferents is too close to that for Ib afferents, we suggest that any physiological explanation of the results must include the participation of Ib afferents and their target interneurons. This possibility is in line with the well-documented evidence that the H-wave is not exclusively monosynaptic and it may be influenced by changes in spinal excitability (Burke et al., 1983, 1984).

## Conclusions

We conclude that plastic changes not only occur in the gain of the H-wave amplitude but also in the H-wave fluctuations as indicated by the differential changes that we found in the FD and CCV in athletes and sedentary subjects. The results obtained presently support the idea that the FD in the H-wave amplitude fluctuations as well as the CCV are useful biomarkers for the evaluation of plastic changes in the motor reflex responses in humans. In this context, the current contribution could be of interest for physical therapists, sports medicine clinicians and neurophysiologists.

## Author contributions

JL and EM conceived and designed the research; MV, JS, AG, FS, JL performed experiments, analyzed data and made figures; NH made a statistical analysis and some figures; EM analyzed the cross-covariance (CCV) data; JL and EM made figures and wrote the manuscript. All authors approved the final version of the manuscript.

### Conflict of interest statement

The authors declare that the research was conducted in the absence of any commercial or financial relationships that could be construed as a potential conflict of interest.
